# *Viroelixir* Exhibits Cell-Line-Specific Anticancer Activity and Enhances Chemosensitivity in Oral Squamous Cell Carcinoma: Associated Changes in Apoptosis, Redox Homeostasis, and Oncogenic Signaling

**DOI:** 10.3390/ijms27198825

**Published:** 2026-10-02

**Authors:** Manal Dahdah, Mahmoud Rouabhia, Hawraa Issa, Lionel Loubaki, Latifa Koussih, Mikhlid H. Almutairi, Abdelhabib Semlali

**Affiliations:** 1 GREB Research Group, Faculty of Dentistry, Laval University, Québec, QC G1V 0A6, Canada; manal.dahdah.1@ulaval.ca (M.D.); mahmoud.rouabhia@fmd.ulaval.ca (M.R.); 2 DREAM-CV Lab, Centre for Outcomes Research and Evaluation, Research Institute of the McGill University Health Centre, Montreal, QC H4A 3J1, Canada; hawraaissa@hotmail.com; 3 Héma-Québec, 1070 Avenue des Sciences-de-la-Vie, Québec, QC G1V 5C3, Canada; lionel.loubaki@hema-quebec.qc.ca; 4 Département des Sciences Expérimentales, Université de Saint-Boniface, Winnipeg, MB R2H 3B8, Canada; lkoussih@ustboniface.ca; 5 Zoology Department, College of Science, King Saud University, P.O. Box 2455, Riyadh 11451, Saudi Arabia; malmutairi@ksu.edu.sa

**Keywords:** oral squamous cell carcinoma, *Viroelixir*, apoptosis, autophagy, oxidative stress, chemosensitization

## Abstract

Oral squamous cell carcinoma (OSCC) remains a major global health burden despite advances in surgery, radiotherapy, and chemotherapy. The development of novel therapeutic agents with improved efficacy and reduced toxicity is therefore urgently needed. This study investigated the anticancer potential of *Viroelixir*, a natural formulation derived from green tea and pomegranate extracts and explored its underlying molecular mechanisms in oral cancer cells. Human gingival squamous cell carcinoma cells (Ca9-22), tongue squamous cell carcinoma cells (CAL-27 and SCC-9), and non-tumorigenic oral epithelial cells (GMSM-K) were exposed to serial dilutions of *Viroelixir*. Cell viability, membrane damage, clonogenic survival, apoptosis, autophagy, redox status, migration, protein expression, and responses to cisplatin or 5-fluorouracil (5-FU) were assessed using MTT and LDH assays, crystal-violet staining, flow cytometry, and wound-healing assays. *Viroelixir* preferentially reduced Ca9-22 viability and clonogenic survival while having limited effects on GMSM-K, CAL-27, and SCC-9SCC-9 viability over 24 h of *Viroelixir* exposure. In Ca9-22 cells, *Viroelixir* increased p21, p27, and p53 and reduced Cyclin D1, a profile consistent with inhibition of G1/S progression. It promoted mitochondrial apoptosis, reduced autophagy-associated signals and LC3B expression, increased intracellular ROS and mitochondrial superoxide, depleted glutathione, and reduced wound closure while increasing E-cadherin and reducing fibronectin. *Viroelixir* also reduced phosphorylation of p38, ERK1/2, STAT3, NF-kappaB, and AKT1. In addition, *Viroelixir* enhanced the antiproliferative effects of cisplatin and 5-fluorouracil. *Viroelixir* exhibits a pronounced cell-line-specific anticancer response, with Ca9-22 gingival carcinoma cells displaying substantially greater sensitivity than the other oral cell models tested. This response is associated with coordinated changes in cell-cycle regulatory proteins, mitochondrial apoptosis, autophagy-associated signals, redox homeostasis, and oncogenic signaling. *Viroelixir* also enhances the antiproliferative effects of cisplatin and 5-FU, supporting further investigation of this formulation in OSCC. These findings support its potential development as a novel adjuvant therapeutic agent for gingival squamous cell carcinoma management.

## Simple Summary

Oral squamous cell carcinoma (OSCC) is one of the most aggressive cancers of the head and neck and is frequently associated with poor prognosis, high recurrence rates, and resistance to chemotherapy. Therefore, the development of safer and more effective therapeutic strategies remains an important clinical challenge. In this study, we investigated the anticancer activity of *Viroelixir*, a natural polyphenol-rich formulation derived from green tea and pomegranate extracts. We demonstrate that *Viroelixir* exerts a pronounced and reproducible antiproliferative effect in Ca9-22 gingival squamous cell carcinoma cells, whereas normal oral epithelial cells and the tongue squamous cell carcinoma lines CAL-27 and SCC-9 show substantially lower acute sensitivity. In Ca9-22 cells, this response is associated with changes in cell-cycle regulatory proteins, mitochondrial apoptosis, autophagy-associated signals, oxidative stress, wound closure, EMT-associated markers, and multiple oncogenic signaling pathways, including protein kinase B (AKT1), extracellular signal-regulated kinases 1/2 (ERK1/2), signal transducer and activator of transcription 3 (STAT3), nuclear factor kappa B (NF-κB), and p38 mitogen-activated protein kinase (p38 MAPK). *Viroelixir* also enhances the antiproliferative activity of cisplatin and 5-fluorouracil, suggesting its potential use as a chemosensitizing agent. These findings identify *Viroelixir* as a promising multitarget natural therapeutic candidate that may complement conventional treatments for oral squamous cell carcinoma.

## 1. Introduction

Oral squamous cell carcinoma (OSCC) is one of the most common malignancies of the head and neck region and remains a major global health challenge [1]. More than 90% of oral cancers are classified as OSCC, a disease characterized by aggressive local invasion, frequent recurrence, and poor survival, particularly when diagnosed at advanced stages [1]. In Canada alone, approximately 8100 new cases of head and neck cancer and more than 2000 related deaths were estimated in 2024 [2]. Despite considerable advances in surgery, radiotherapy, and chemotherapy, the prognosis of patients with advanced OSCC remains poor. Furthermore, these conventional treatment modalities are often associated with severe adverse effects that impair patients’ quality of life and may compromise treatment adherence [3,4,5,6]. Therefore, the development of safer and more effective therapeutic strategies remains an important clinical priority. Of relevance to natural multitarget therapeutic approaches, treatment-induced alterations in tumor-derived EVs may provide an additional mechanism through which anticancer agents influence intercellular communication within the tumor microenvironment [7,8]. This emerging concept warrants further investigation in OSCC, including assessment of whether bioactive formulations can modify the molecular cargo and biological activity of cancer cell-derived EVs.

Natural products have emerged as valuable sources of anticancer agents due to their broad biological activities and favorable safety profiles [9,10,11]. Epidemiological studies indicate that a large proportion of cancer patients use complementary and alternative medicine alongside conventional therapies [12,13]. Unlike many conventional drugs that target a single molecular pathway, phytochemicals often exert pleiotropic effects by simultaneously modulating multiple cellular processes involved in tumor development and progression [14]. Among these processes, dysregulation of cell-cycle regulation, apoptosis, autophagy, oxidative stress, and intracellular signaling pathways plays a central role in OSCC pathogenesis, making them attractive therapeutic targets [15,16].

Green tea and pomegranate are among the most extensively investigated plant-derived products for cancer prevention and treatment. Green tea contains high concentrations of catechins, particularly epigallocatechin-3-gallate (EGCG), which exhibit antiproliferative, pro-apoptotic, antioxidant, and chemosensitizing properties in various cancer models [17,18,19,20,21,22,23]. Similarly, pomegranate is rich in polyphenols, hydrolysable tannins, anthocyanins, and punicalagin, compounds known to regulate multiple signaling pathways involved in cell proliferation, apoptosis, inflammation, and oxidative stress [24,25]. Growing evidence suggests that these bioactive compounds can interfere with tumor progression through complementary and synergistic mechanisms of action [26,27]. Based on these observations, we investigated *Viroelixir*, a polyphenol-rich formulation combining green tea and pomegranate extracts. We hypothesized that this formulation could exert anticancer effects associated with the modulation of multiple cellular processes involved in oral carcinogenesis. Therefore, the present study aimed to evaluate the cell-line-specific response to *Viroelixir* in oral cancer and non-tumorigenic oral cell models and to characterize its effects on cell proliferation, cell-cycle regulatory proteins, apoptosis, autophagy-associated signals, redox homeostasis, wound closure, and major signaling proteins associated with OSCC.

## 2. Results

### 2.1. Viroelixir Induces a Pronounced Cell-Line-Specific Response in Ca9-22 Gingival Carcinoma Cells

**Figure 1:**
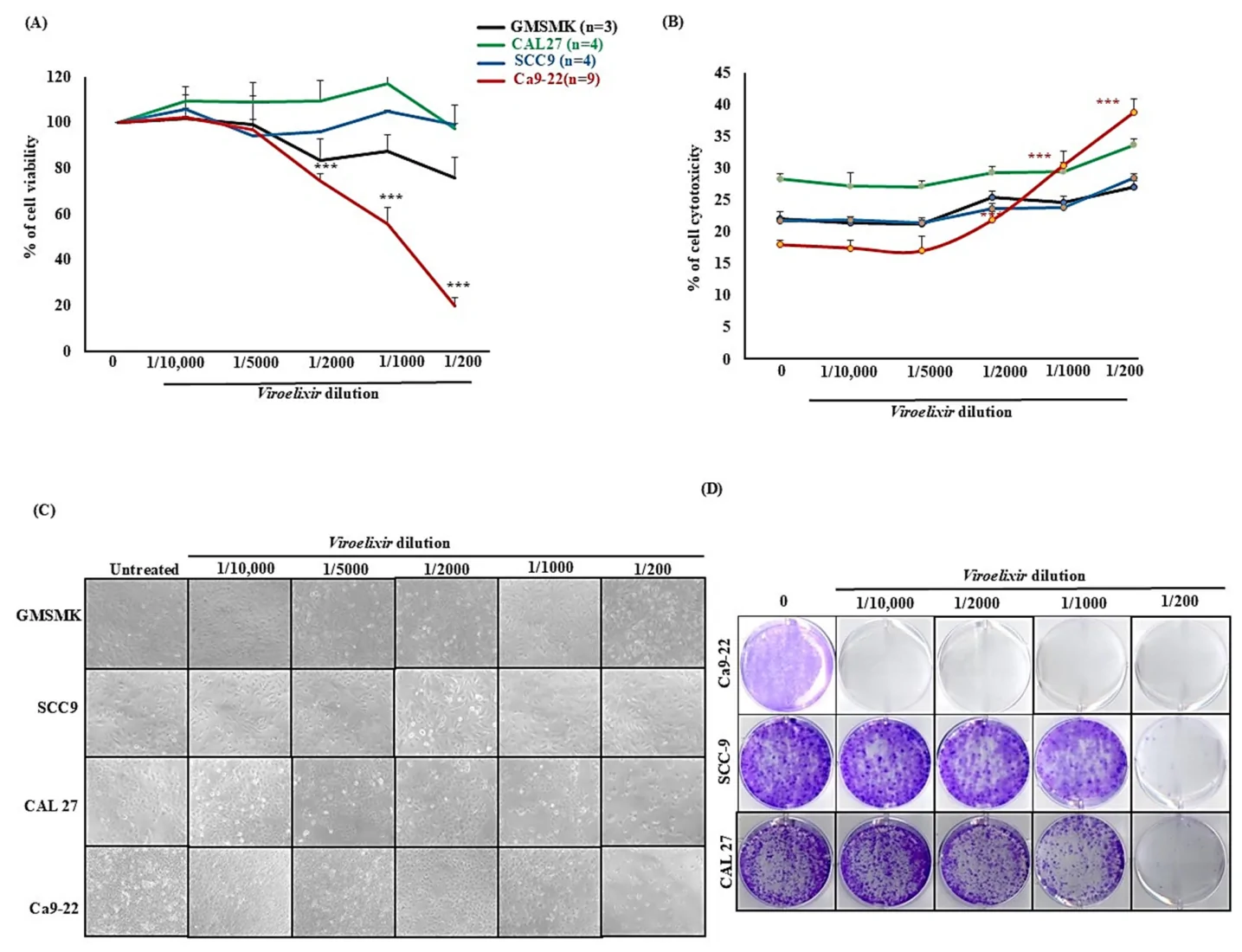
*Viroelixir* differentially affects viability, membrane integrity, morphology, and clonogenic survival in oral cell lines. (**A**) Viability of GMSM-K, CAL-27, SCC-9SCC-9, and Ca9-22 cells after 24 h of exposure to serial *Viroelixir* dilutions, measured by MTT assay. The number of independent experiments was GMSM-K, n = 3; CAL-27, n = 4; SCC-9SCC-9, n = 4; and Ca9-22, n = 9. Each independent experiment was performed with four technical replicates per experimental condition. (**B**) LDH release after 24 h as an index of membrane damage. (**C**) Representative phase-contrast images of cell morphology. (**D**) Representative crystal-violet-stained colonies after 14 days. Data are mean +/− SD. Significance symbols denote comparisons with the untreated control: *** *p* < 0.001.

To determine the anticancer potential of *Viroelixir*, oral squamous cell carcinoma cell lines (Ca9-22, SCC-9SCC-9, and CAL-27) and normal oral epithelial cells (GMSM-K) were exposed to increasing concentrations of *Viroelixir* (1/10,000–1/200 dilution) for 24 h, and cell viability was assessed using the MTT assay. As shown in Figure 1A, *Viroelixir* induced a markedly cell-line-dependent antiproliferative response. Ca9-22 gingival squamous carcinoma cells exhibited pronounced concentration-dependent sensitivity, whereas SCC-9SCC-9 and CAL-27 tongue carcinoma cells remained largely unaffected after 24 h of treatment. In addion, *Viroelixir* treatment exhibited a progressive decrease in viability from 97.0 ± 4.2% at the 1/5000 dilution to 74.4 ± 3.4% at the 1/2000 dilution (*p* < 0.001), 56.0 ± 7.1% at the 1/1000 dilution (*p* = 0.047), and 19.9 ± 3.6% at the 1/200 dilution (*p* < 0.001). In contrast, SCC-9SCC-9 and CAL-27 tongue carcinoma cells remained largely unaffected, maintaining viability levels close to those of untreated controls across all tested dilutions. At the 1/200 dilution, SCC-9SCC-9 and CAL-27 cells retained viabilities of 99.3 ± 0.5% and 97.6 ± 10.2%, respectively. Normal GMSM-K cells also displayed limited sensitivity to *Viroelixir*, with viability remaining at 75.9 ± 8.8% at the 1/200 dilution, indicating a markedly greater sensitivity of Ca9-22 cells compared with the other oral cell models tested.

The cytotoxic activity of *Viroelixir* was further investigated using the LDH release assay. As illustrated in Figure 1B, treatment induced a concentration-dependent increase in LDH release in oral cancer cells, particularly in Ca9-22 cells. Cytotoxicity increased from 18.0 ± 0.7% in untreated cells to 30.5 ± 2.3% and 38.8 ± 2.2% following treatment with the 1/1000 and 1/200 dilutions, respectively (*p* = 0.0023 and *p* < 0.001). A significant increase was already detectable at the 1/2000 dilution (21.9 ± 1.3%, *p* = 0.037). In contrast, only modest changes were observed in normal GMSM-K cells, where cytotoxicity remained below 30% at the 1/200 dilution (27.1 ± 1.3%). Similarly, SCC-9SCC-9 and CAL-27 cells exhibited limited LDH release compared with Ca9-22 cells. These findings indicate that *Viroelixir* preferentially induces cytotoxicity in Ca9-22 oral cancer cells while exerting minimal effects on normal oral epithelial cells.

Microscopic examination revealed dilution-dependent reductions in cell density and morphological alterations (Figure 1C). These changes were most pronounced in Ca9-22 cells, whereas GMSM-K, SCC-9SCC-9, and CAL-27 cells exhibited only minor morphological changes after 24 h of treatment, consistent with the MTT and LDH results.

To assess the long-term effect of *Viroelixir* on tumorigenic potential, colony formation assays were performed. *Viroelixir* markedly reduced the clonogenic capacity of oral cancer cells in a dose-dependent manner. Treatment with the highest concentration almost completely abolished colony formation in Ca9-22 cells, whereas SCC-9SCC-9 and CAL-27 cells exhibited a moderate reduction in colony number and size.

Interestingly, SCC-9SCC-9 cells showed a more pronounced reduction in colony formation at the highest *Viroelixir* concentration than would have been predicted from the 24 h MTT assay. This apparent difference likely reflects the distinct biological endpoints assessed by the two assays: whereas MTT measures short-term metabolic activity, the clonogenic assay evaluates the long-term reproductive capacity of surviving cells. Thus, SCC-9SCC-9 cells may retain metabolic activity shortly after *Viroelixir* exposure while exhibiting impaired subsequent colony-forming ability.

### 2.2. Viroelixir Modulates Cell-Cycle Regulatory Proteins in Ca9-22 Cells

**Figure 2:**
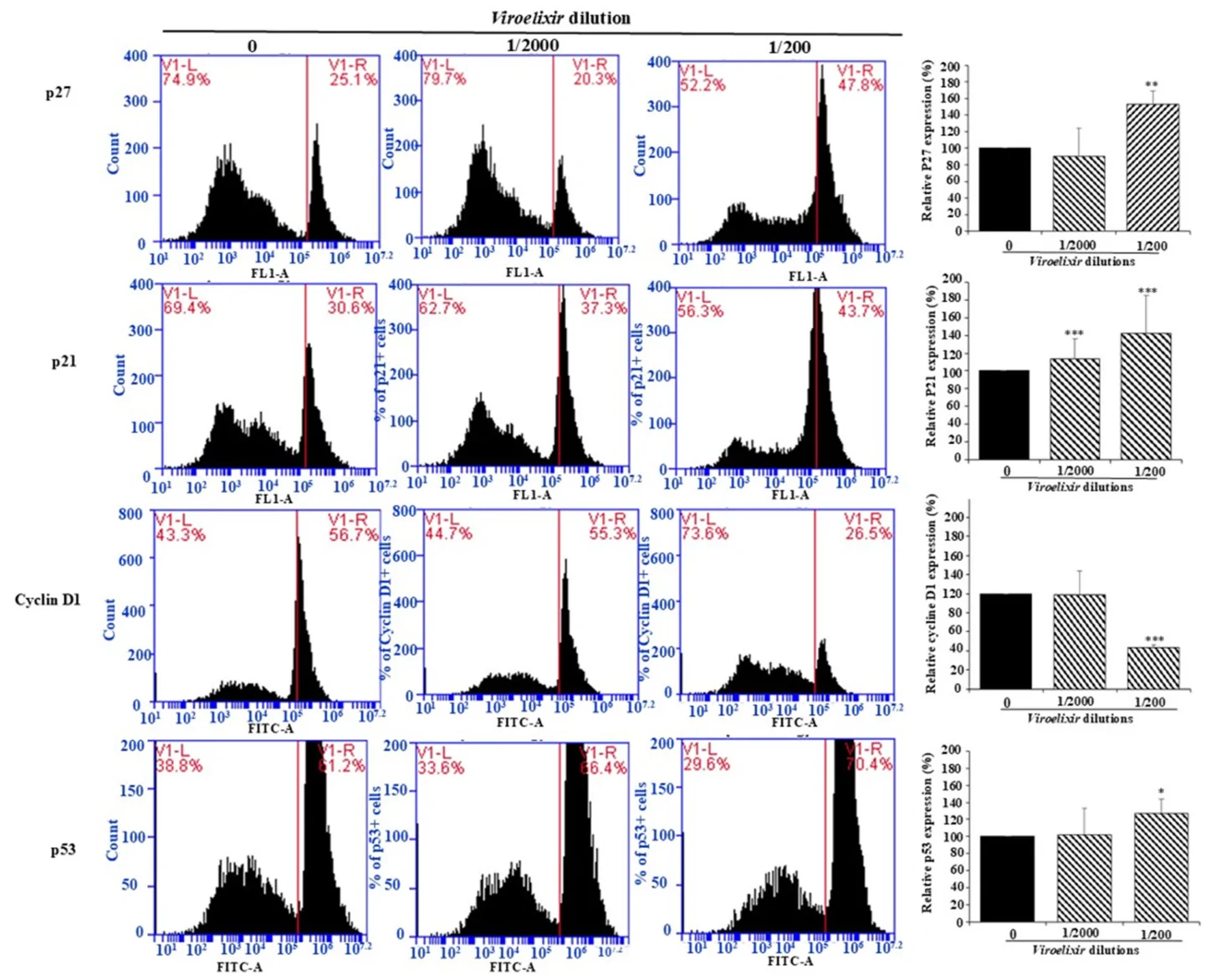
*Viroelixir* modulates cell-cycle regulatory proteins in Ca9-22 cells. Representative flow-cytometry histograms and quantitative analysis of p27, p21, Cyclin D1, and p53 after 24 h of exposure to *Viroelixir* (0, 1/2000, or 1/200). The red vertical lines indicate the fluorescence threshold, established using the negative control, to distinguish negative from positive cell populations. Values are normalized to untreated controls and presented as mean ± SD. * *p* < 0.05, ** *p* < 0.01, and *** *p* < 0.001 versus untreated cells.

To further elucidate the mechanisms underlying the antiproliferative activity of *Viroelixir*, the expression of key cell-cycle regulatory proteins was analyzed by flow cytometry following treatment of Ca9-22 oral cancer cells. As shown in Figure 2, *Viroelixir* significantly modulated the expression of several key regulators involved in cell-cycle progression. Treatment with the 1/200 dilution significantly increased the expression of the cyclin-dependent kinase inhibitors p21 and p27, reaching 158.7 ± 42.1% and 153.3 ± 16.1% of control levels, respectively (*p* < 0.001 and *p* = 0.0039). Likewise, p53 expression increased to 127.2 ± 16.2% of control values following treatment with the 1/200 dilution (*p* < 0.05). In contrast, Cyclin D1 expression was markedly reduced to 43.4 ± 2.7% of control levels at the same concentration (*p* < 0.001). No significant changes were observed at the 1/2000 dilution for p27, Cyclin D1, or p53. Representative histograms confirmed these findings, showing an increase in the proportion of p53-, p21-, and p27-positive cells, accompanied by a marked reduction in Cyclin D1-positive cells following *Viroelixir* treatment. Collectively, these findings demonstrate that *Viroelixir* induces coordinated changes in key cell-cycle regulatory proteins in Ca9-22 cells, characterized by increased p53, p21, and p27 expression and decreased Cyclin D1 expression. This molecular profile is consistent with impaired G1/S progression; however, direct demonstration of cell-cycle phase redistribution would require DNA-content analysis.

### 2.3. Viroelixir Induces Mitochondrial-Mediated Apoptosis in Oral Cancer Cells

**Figure 3:**
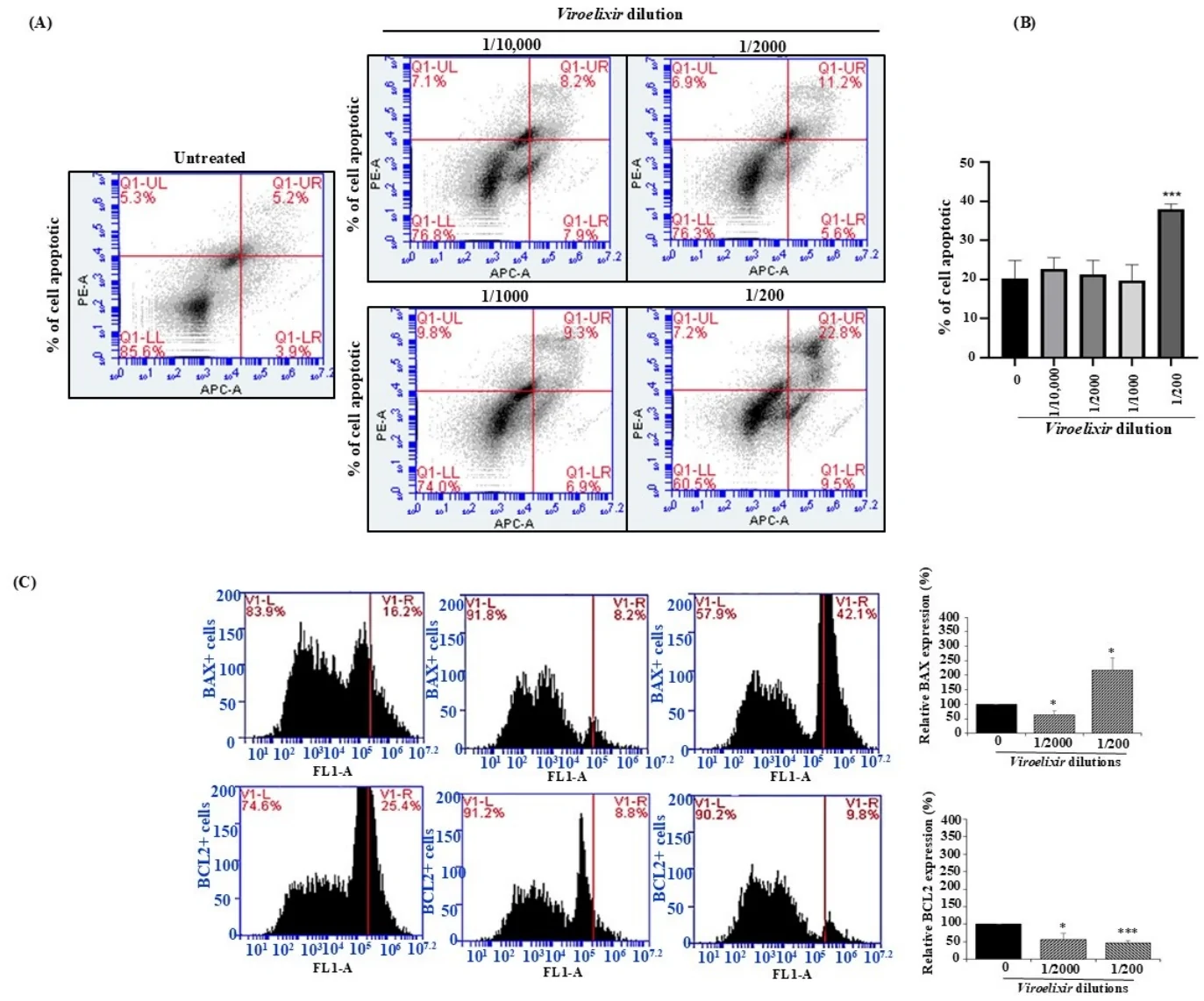

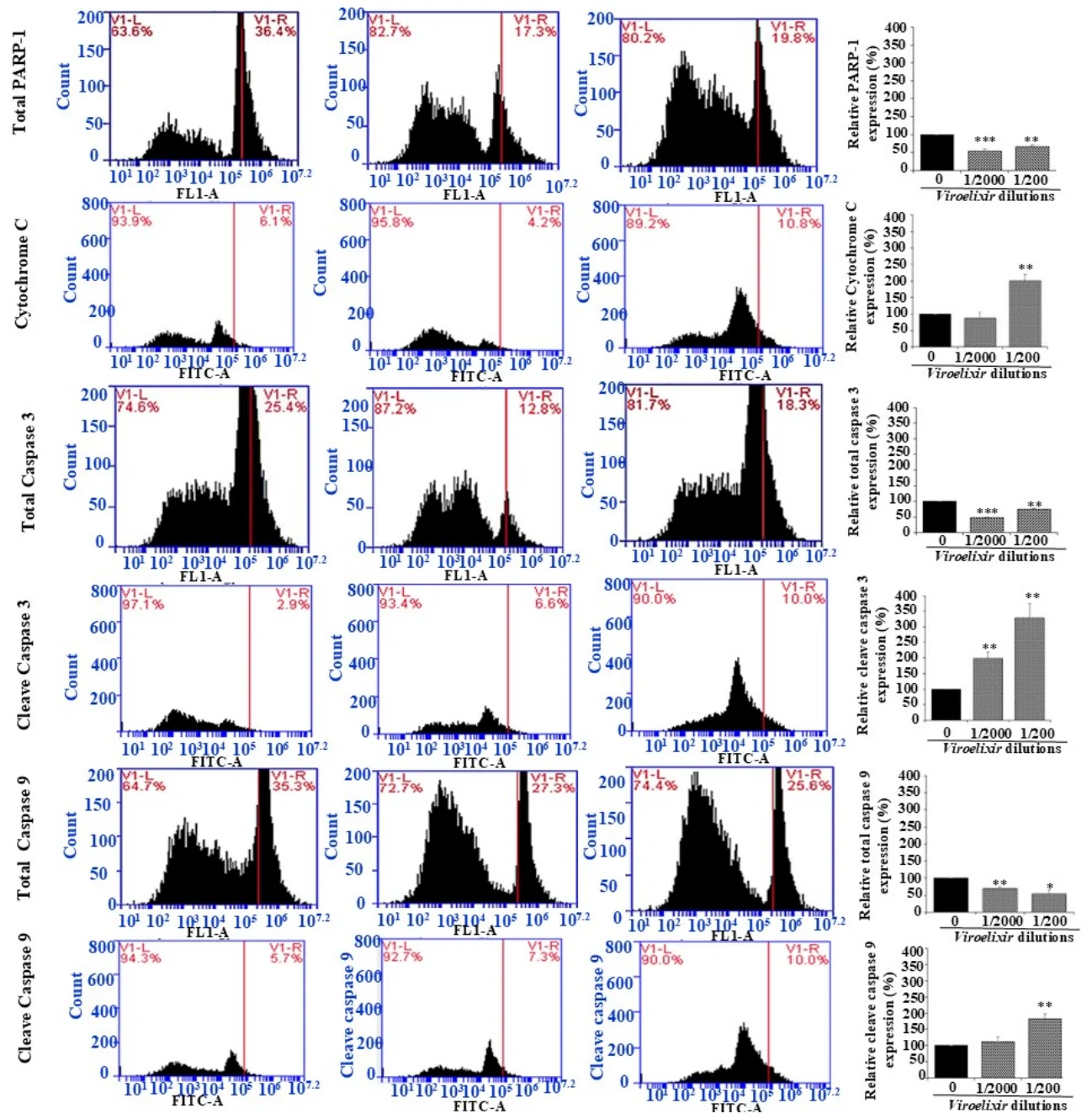
*Viroelixir* activates mitochondrial apoptosis in Ca9-22 cells. (**A**) Representative Annexin V/PI dot plots after 24 h of exposure to *Viroelixir* (0, 1/10,000, 1/2000, 1/1000, or 1/200). (**B**) Quantification of apoptotic cells across the dilution series. (**C**) Representative flow-cytometry histograms and normalized quantification of Bax, Bcl-2, total PARP-1, cytochrome c, total caspase-3, cleaved caspase-3, total caspase-9, and cleaved caspase-9 after exposure to 0, 1/2000, or 1/200 *Viroelixir*. The red vertical lines indicate the fluorescence threshold, established using the negative control, to distinguish negative from positive cell populations. Data means +/− SD. * *p* < 0.05, ** *p* < 0.01, and *** *p* < 0.001 versus untreated cells.

To determine whether apoptosis contributed to the antiproliferative effect of *Viroelixir*, Ca9-22 cells were exposed to serial dilutions for 24 h and analyzed by Annexin V/PI flow cytometry (Figure 3A, B). The quantified apoptotic population remained close to the untreated level at 1/10,000, 1/2000, and 1/1000, but increased to approximately 38% at 1/200 compared with approximately 20% in untreated cultures (*p* < 0.001). Thus, a significant apoptotic response was detected principally at the highest tested concentration.

The mitochondrial apoptosis pathway was then examined (Figure 3C). At 1/2000, Bax expression initially decreased to approximately 64% of the control (*p* < 0.05) at the 1/2000 dilution before increasing to approximately 218% at the 1/200 dilution (*p* < 0.05). Bcl-2 decreased to approximately 56% and 47% of the control at 1/2000 and 1/200, respectively. At 1/200, cytochrome c increased to approximately 200% of the control, while total PARP-1 decreased at both dilutions. Total caspase-3 and caspase-9 abundance decreased, whereas their cleaved forms increased: cleaved caspase-3 rose to approximately 200% and 330% of the control at 1/2000 and 1/200, and cleaved caspase-9 rose most clearly at 1/200 to approximately 180%. Collectively, the increase in apoptosis, cytochrome c, cleaved caspase-9, and cleaved caspase-3, together with increased Bax and reduced Bcl-2 at 1/200, supports activation of the intrinsic mitochondrial apoptotic pathway at the highest *Viroelixir* concentration. Because only total PARP-1 expression was evaluated, these data indicate a reduction in total PARP-1 abundance rather than PARP cleavage.

### 2.4. Viroelixir Reduces Autophagy-Associated Signals in Ca9-22 Cells

**Figure 4:**
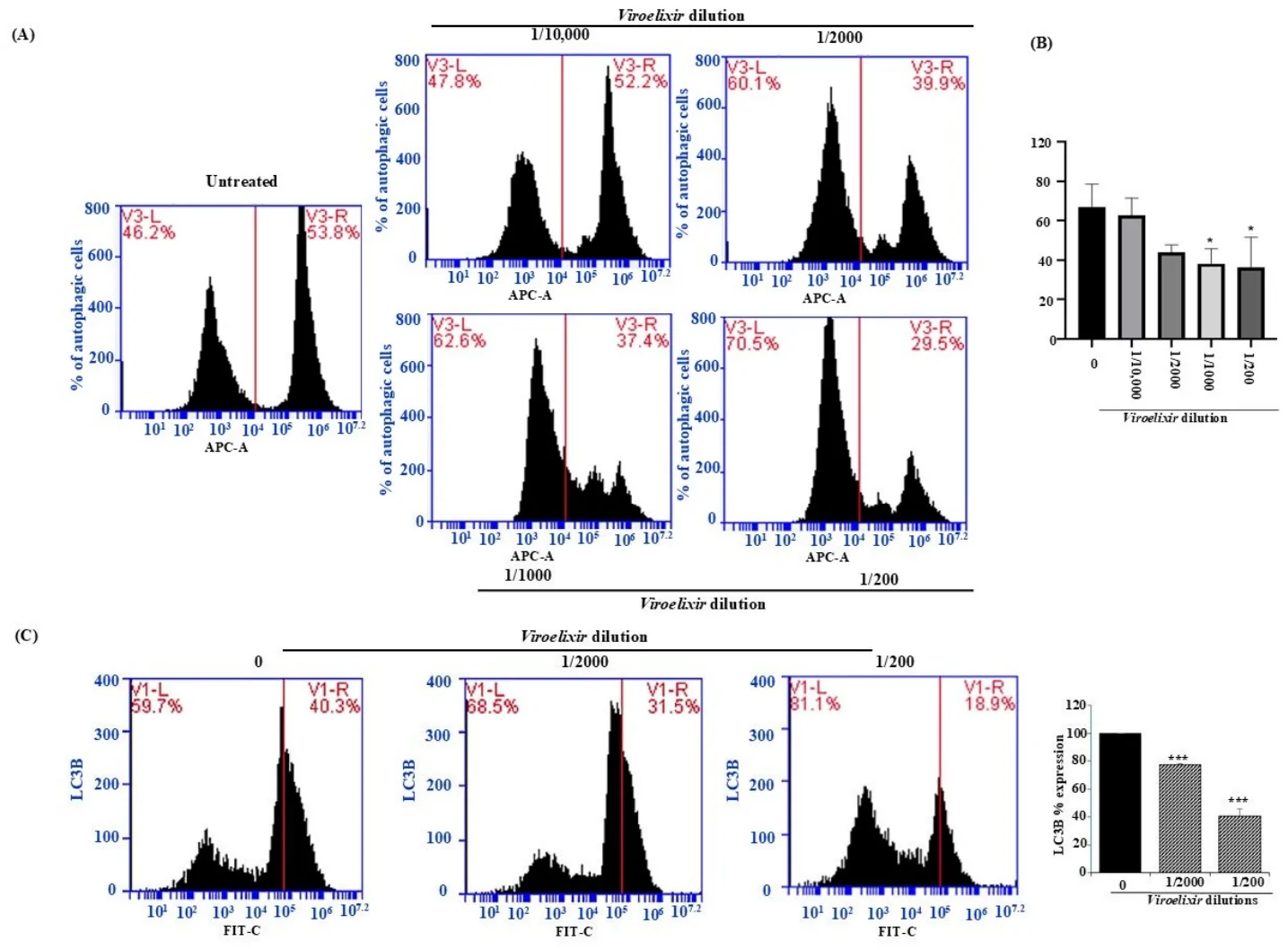
*Viroelixir* reduces autophagy-associated readouts in Ca9-22 cells. (**A**) Representative flow-cytometry histograms of fluorescent autophagy staining after 24 h of exposure to the indicated *Viroelixir* dilutions. (**B**) Quantification of autophagy-positive cells across the dilution series. (**C**) Representative histograms and normalized quantification of LC3B expression at 0, 1/2000, and 1/200. The red vertical lines indicate the fluorescence threshold, established using the negative control, to distinguish negative from positive cell populations. Data are mean +/− SD. * *p* < 0.05 and *** *p* < 0.001 versus untreated cells. These endpoints indicate altered autophagy-associated signals but do not directly measure autophagic flux.

Autophagy-associated fluorescence decreased with increasing *Viroelixir* exposure in Ca9-22 cells (Figure 4A, B). The autophagy-positive population fell from 53.8% in untreated cells to 39.9% and 29.5% at 1/2000 and 1/200, respectively; the full quantitative series also showed significant reductions at 1/1000 and 1/200. LC3B expression decreased to 77.3 +/− 0.7% and 40.8 +/− 5.0% of the control at 1/2000 and 1/200 (both *p* < 0.001; Figure 4C). These findings demonstrate that *Viroelixir* reduces autophagy-associated fluorescence and LC3B expression in Ca9-22 cells. These results indicate a modulation of autophagy-associated processes following *Viroelixir* treatment; however, because autophagic flux was not directly assessed, they do not distinguish between reduced autophagosome formation and altered autophagosome turnover or clearance.

### 2.5. Viroelixir Modulates Oxidative Stress and Mitochondrial Redox Homeostasis in Oral Cancer Cells

**Figure 5:**
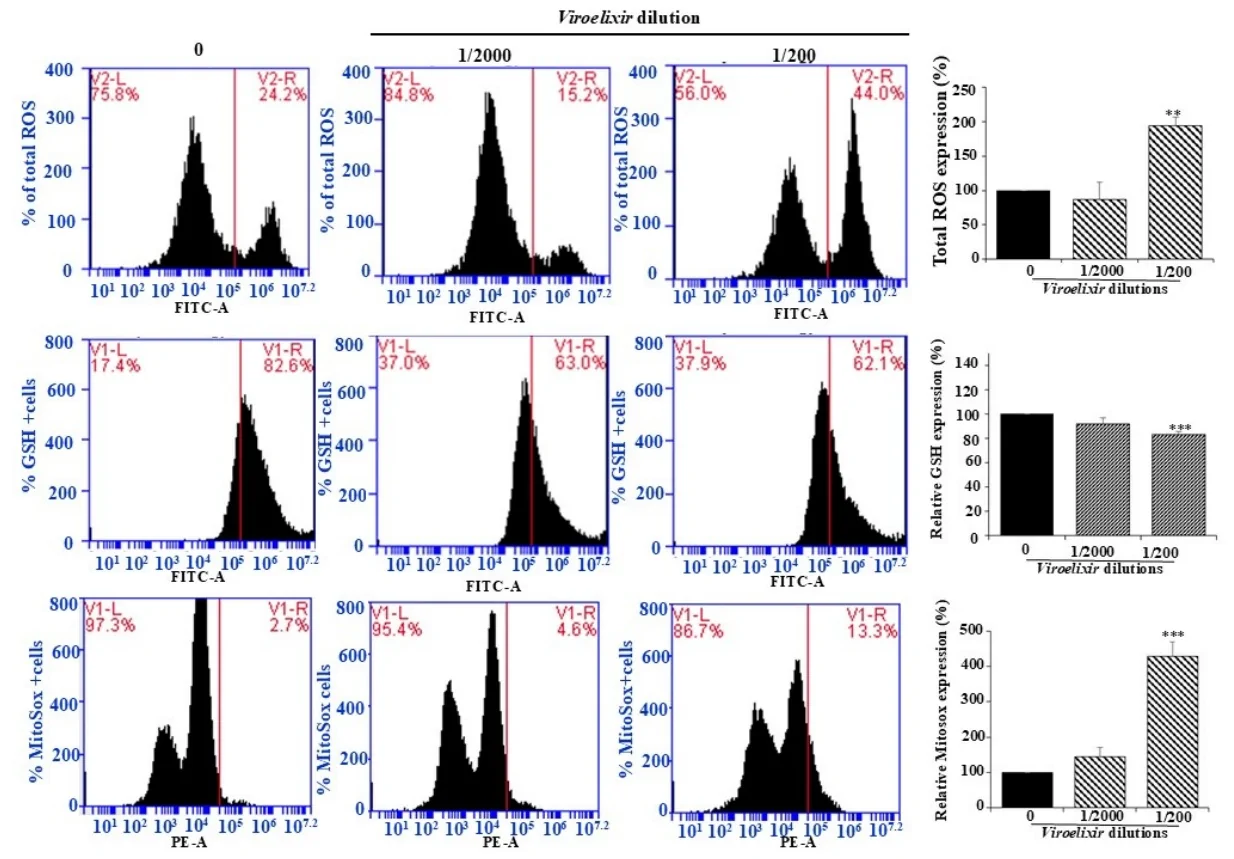
*Viroelixir* disrupts redox homeostasis in Ca9-22 cells. Representative flow-cytometry histograms and quantitative analyses of (**top**) total intracellular ROS, (**middle**) intracellular glutathione (GSH), and (**bottom**) mitochondrial superoxide detected with MitoSOX after 24 h of exposure to *Viroelixir* (0, 1/2000, or 1/200). The red vertical lines indicate the fluorescence threshold, established using the negative control, to distinguish negative from positive cell populations. Quantitative values are shown as positive-cell percentages for total ROS and as expression normalized to untreated controls for GSH and MitoSOX. Data are mean +/− SD. ** *p* < 0.01 and *** *p* < 0.001 versus untreated cells. The number of independent experiments and statistical test should be specified before submission.

*Viroelixir* altered redox homeostasis in Ca9-22 cells (Figure 5). Total ROS-positive cells increased from 22.2 +/− 2.8% in untreated cultures to 42.9 +/− 2.1% at 1/200 (*p* < 0.01), whereas the small change at 1/2000 was not significant. Relative intracellular GSH decreased from 100% in untreated cells to 91.6 +/− 5.1% and 83.1 +/− 2.6% at 1/2000 and 1/200, respectively, with a significant reduction at 1/200 (*p* < 0.001). Mitochondrial superoxide increased from 2.7% MitoSOX-positive cells in untreated cultures to 4.6% at 1/2000 and 13.3% at 1/200; normalized MitoSOX expression reached approximately 145% and 425% of the control, respectively, with a significant increase at 1/200 (*p* < 0.001). Collectively, these findings indicate that *Viroelixir* increases both intracellular and mitochondrial oxidative stress while simultaneously depleting intracellular GSH, particularly at the highest concentration tested.

### 2.6. Viroelixir Reduces Wound Closure and Alters EMT-Associated Markers in Ca9-22 Cells

**Figure 6:**
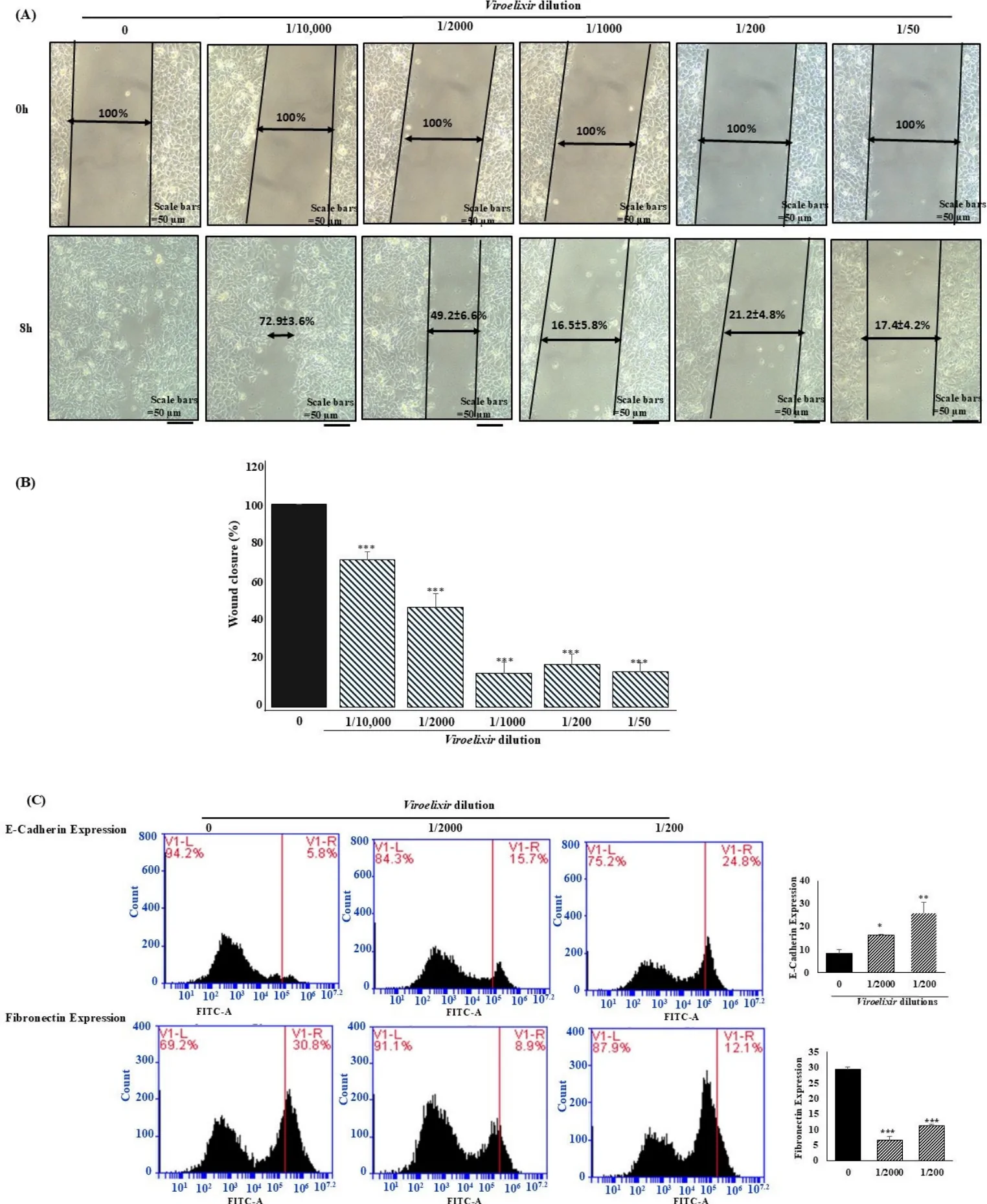
*Viroelixir* reduces wound closure and modulates EMT-associated proteins in Ca9-22 cells. (**A**) Representative images at 0 and 8 h after scratch formation in cultures exposed to the indicated *Viroelixir* dilutions. (**B**) Quantitative analysis of the residual wound width after 8 h of *Viroelixir* treatment, expressed as a percentage of the initial wound width at 0 h. The red vertical lines indicate the fluorescence threshold, established using the negative control, to distinguish negative from positive cell populations. Data are presented as mean ± SD from four independent experiments. Statistical significance was determined by Student’s t-test compared with the untreated control. *** *p* < 0.001. (**C**) Representative flow-cytometry histograms and quantitative analyses of E-cadherin and fibronectin after 24 h of exposure to 0, 1/2000, or 1/200 *Viroelixir*. Data are mean +/− SD. * *p* < 0.05, ** *p* < 0.01, and *** *p* < 0.001 versus untreated cells. Scale bars = 50 µm.

*Viroelixir* significantly reduced wound closure in Ca9-22 cells compared with untreated controls (Figure 6A,B). Quantitative analysis of the wound area at 0 and 8 h confirmed a concentration-dependent reduction in wound closure following *Viroelixir* treatment. These findings indicate impaired wound closure under *Viroelixir* treatment. However, because wound closure is influenced by both cell migration and cell proliferation/viability, these results should not be interpreted as definitive evidence of a migration-specific effect. Percentage of wound closure was calculated by the formula

Wound closure (%) = [(Area at 0 h − Area at 8 h)/Area at 0 h] × 100.

Consistent with this phenotype, *Viroelixir* also altered EMT-associated proteins (Figure 6C). E-cadherin-positive cells increased from 5.8% in untreated cultures to 15.7% and 24.8% at 1/2000 and 1/200, respectively. Fibronectin-positive cells decreased from 30.8% to 8.9% and 12.1%, respectively. The quantified data confirmed significant E-cadherin induction and fibronectin reduction, consistent with a shift toward a less mesenchymal phenotype.

### 2.7. Viroelixir Modulates Multiple Signaling Pathways Involved in Oral Cancer Progression

**Figure 7:**
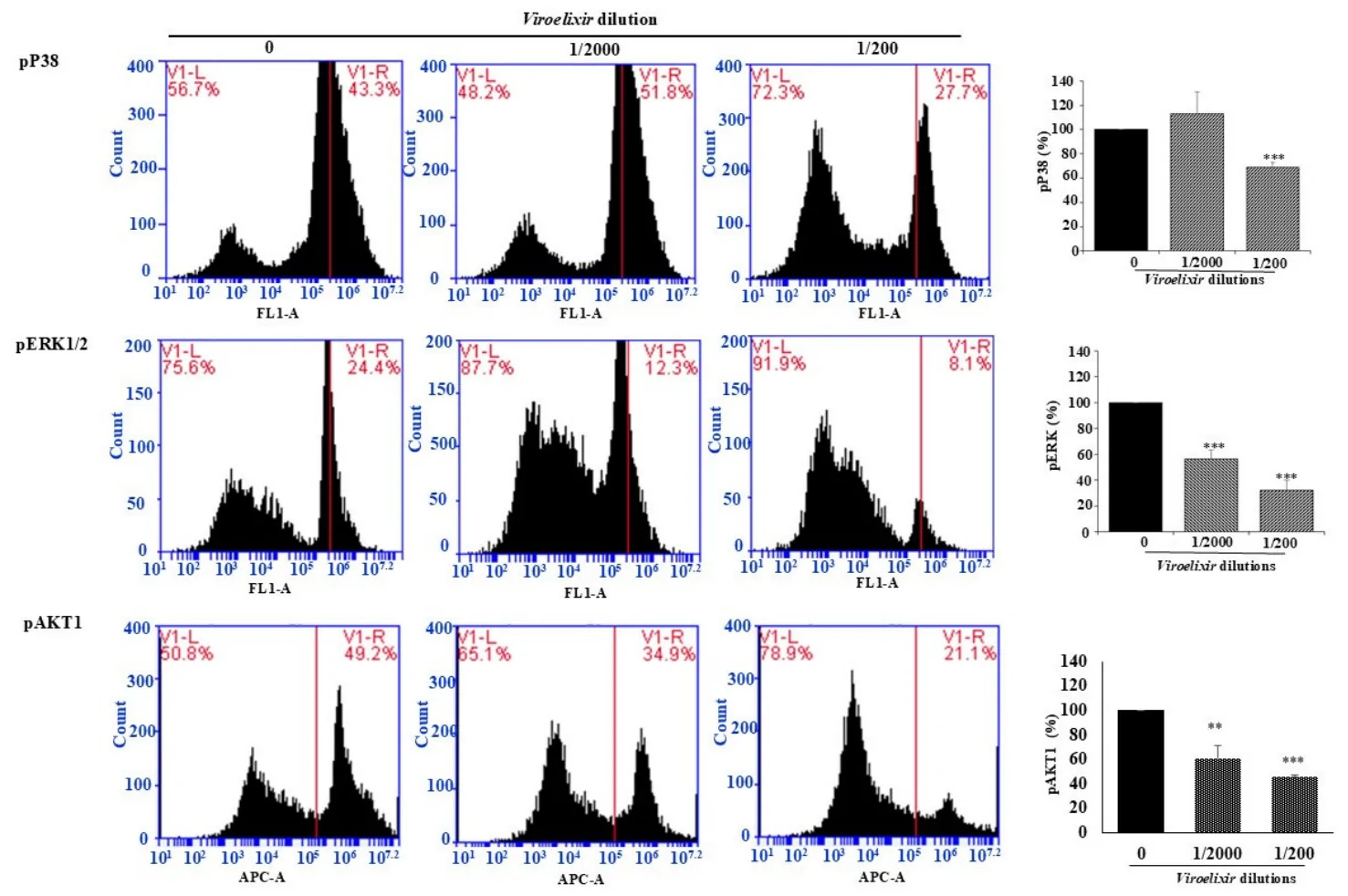

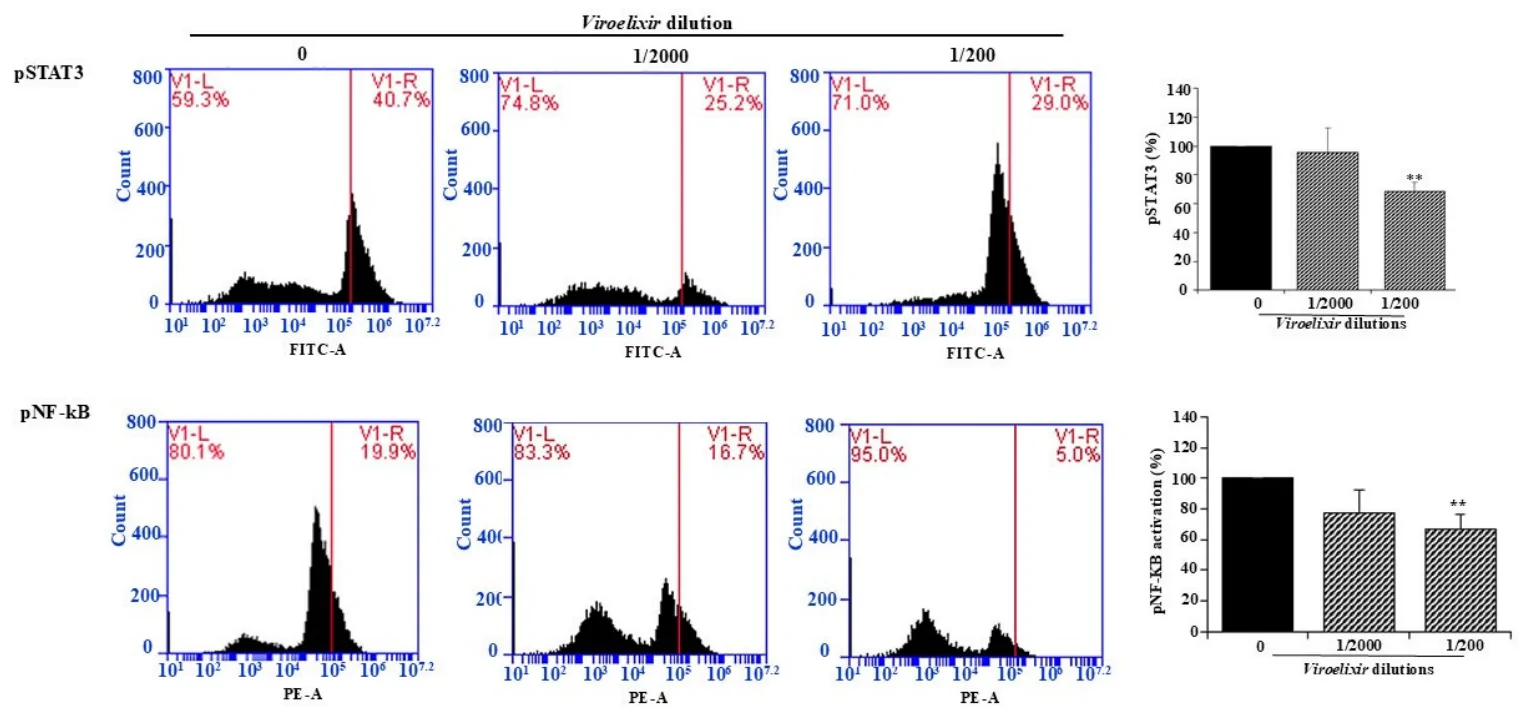
*Viroelixir* attenuates oncogenic and inflammatory signaling in Ca9-22 cells. Representative flow-cytometry histograms and quantitative analyses of phosphorylated p38 MAPK, ERK1/2, AKT1, STAT3, and NF-κB p65 after 24 h of exposure to *Viroelixir* (0, 1/2000, or 1/200). p38, ERK1/2, STAT3, and NF-κB p65 values are shown relative to untreated controls; AKT1 is shown as the percentage of phospho-AKT1-positive cells. Data are mean +/− SD. ** *p* < 0.01, and *** *p* < 0.001 versus untreated cells. Data from four independent experiments are expressed relative to the corresponding untreated control within each experiment, which was defined as 100%. Consequently, the normalized untreated control has a mean value of 100% and an SD of 0; therefore, no error bar is visible for the control group.

To examine signaling pathways associated with proliferation, survival, inflammation, and stress responses, phosphorylated p38, ERK1/2, AKT1, STAT3, and NF-κB were analyzed in Ca9-22 cells after 24 h (Figure 7). Phosphorylated p38 levels were unchanged at 1/2000 but decreased to 69.1 +/− 3.6% of the control at 1/200 (*p* < 0.001). ERK1/2 phosphorylation decreased to 56.5 +/− 6.7% and 32.3 +/− 7.7% of the control at 1/2000 and 1/200, respectively (both *p* < 0.001). These findings indicate concentration-dependent suppression of ERK1/2 signaling together with inhibition of p38 phosphorylation at the highest *Viroelixir* concentration. Phospho-AKT1-positive cells decreased from approximately 53% in untreated cultures to approximately 33% at 1/2000 and 24% at 1/200 (*p* < 0.05 and *p* < 0.01, respectively; Figure 7). Relative pSTAT3 remained close to control at 1/2000 but decreased to 68.4 +/− 6.5% at 1/200 (*p* < 0.01). Relative phospho- NF-κB decreased to 77.1 +/− 15.3% and 67.0 +/− 9.6% at 1/2000 and 1/200, with significance at 1/200 (*p* < 0.01); the representative positive population decreased from 19.9% to 5.0%. Collectively, these findings demonstrate that *Viroelixir* reduced the levels of phosphorylated AKT1, ERK1/2, STAT3, NF-κB, and p38 as assessed by intracellular phospho-specific flow cytometry, with the most pronounced effects observed at the 1/200 dilution.

### 2.8. Viroelixir Enhances the Antiproliferative Effects of Cisplatin and 5-Fluorouracil in Ca9-22 Cells

**Figure 8:**
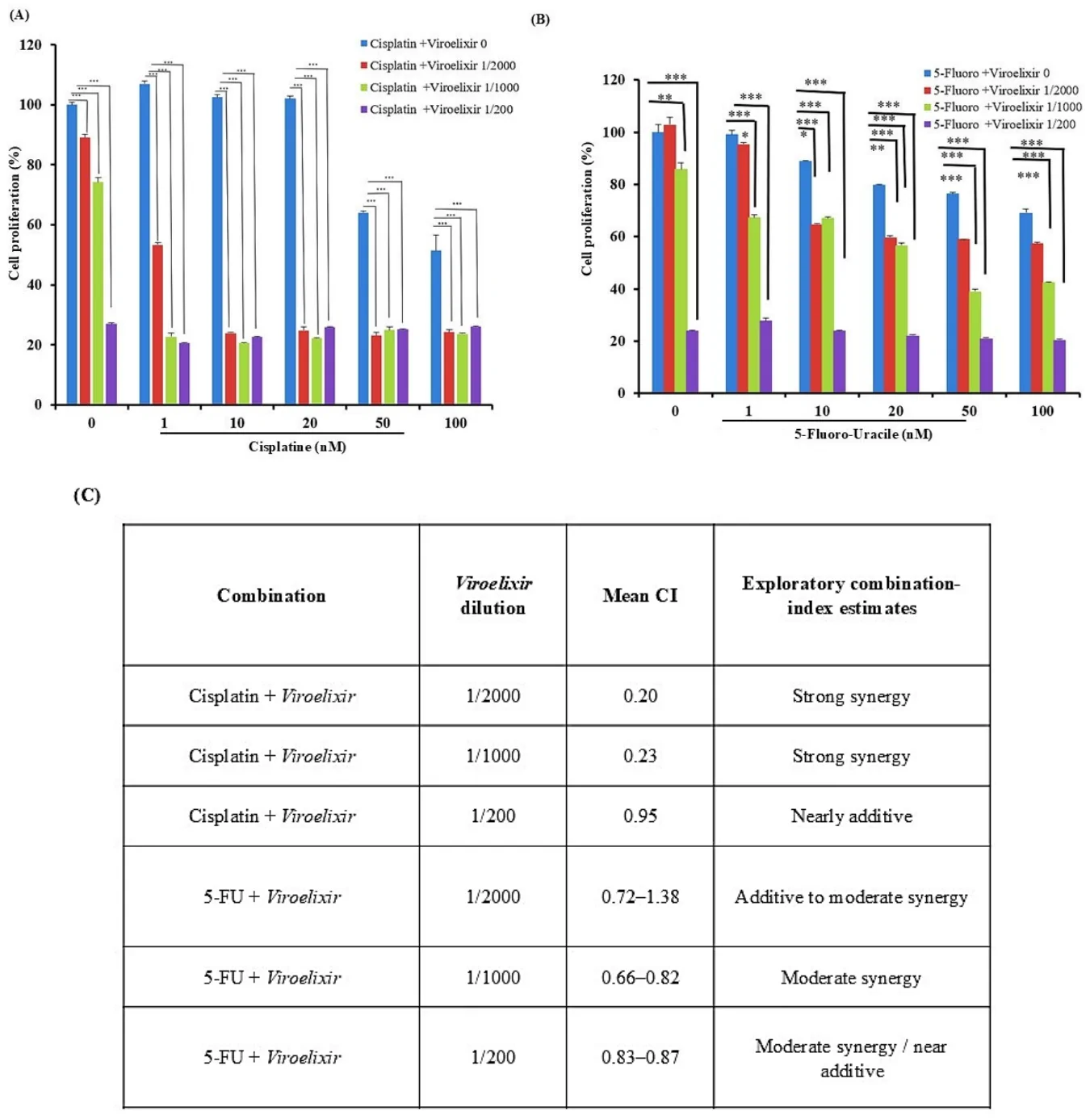
*Viroelixir* enhances the antiproliferative effects of cisplatin and 5-fluorouracil in Ca9-22 cells. (**A**) MTT viability after 24 h of exposure to cisplatin (0–100 nM) combined with *Viroelixir* (0, 1/2000, 1/1000, or 1/200). (**B**) MTT viability after 24 h of exposure to 5-fluorouracil (5-FU; 0–100 micromolar) combined with the same *Viroelixir* dilutions. (**C**) Exploratory combination-index (CI) estimates for *Viroelixir* combined with cisplatin or 5-FU. Because a strict fixed-ratio or comprehensive dose-matrix design was not used, CI values are presented as exploratory interaction estimates and should not be interpreted as definitive evidence of pharmacological synergy. Data are mean +/− SD. * *p* < 0.05, ** *p* < 0.01, and *** *p* < 0.001 for the comparisons indicated.

To determine whether *Viroelixir* could enhance the efficacy of conventional chemotherapeutic agents, oral cancer cells were treated with increasing concentrations of 5-fluorouracil (5-FU) or cisplatin in the presence or absence of *Viroelixir.* Cell viability was subsequently evaluated using the MTT assay. As shown in Figure 8A, treatment with 5-FU alone induced a moderate reduction in cancer cell proliferation. However, the addition of *Viroelixir* significantly enhanced the antiproliferative activity of 5-FU in a concentration-dependent manner. At the lowest *Viroelixir* dilution (1/2000), cell proliferation was reduced compared with 5-FU alone, whereas stronger inhibition was observed at dilutions of 1/1000 and 1/200. The most pronounced effect was detected with the combination of 5-FU and *Viroelixir* at the 1/200 dilution, where cell proliferation decreased to approximately 20–30% of control levels across the different 5-FU concentrations tested. These findings suggest that *Viroelixir* enhances the sensitivity of oral cancer cells to 5-FU treatment. Similarly, *Viroelixir* significantly potentiated the anticancer activity of cisplatin (Figure 8B). While cisplatin alone produced a dose-dependent inhibition of cell proliferation, the addition of *Viroelixir* further enhanced this effect. Combination treatment with cisplatin and *Viroelixir* at 1/1000 and 1/200 dilutions resulted in a marked reduction in cell viability compared with cisplatin alone. At several cisplatin concentrations, cell proliferation was reduced by more than 70–80% following combination treatment, demonstrating a substantial increase in therapeutic efficacy. Exploratory CI estimates were <1 for several *Viroelixir*–cisplatin and *Viroelixir*–5-FU combinations, suggesting potentially favorable drug interactions. However, because the experiments were not performed using a strict fixed-ratio or comprehensive dose-matrix design, these CI values should be considered exploratory and cannot establish formal pharmacological synergy. The principal finding of these experiments is therefore that *Viroelixir* enhances the antiproliferative effects of cisplatin and 5-FU in Ca9-22 cells.

### 2.9. Proposed Mechanism Underlying the Anticancer Activity of Viroelixir

**Figure 9:**
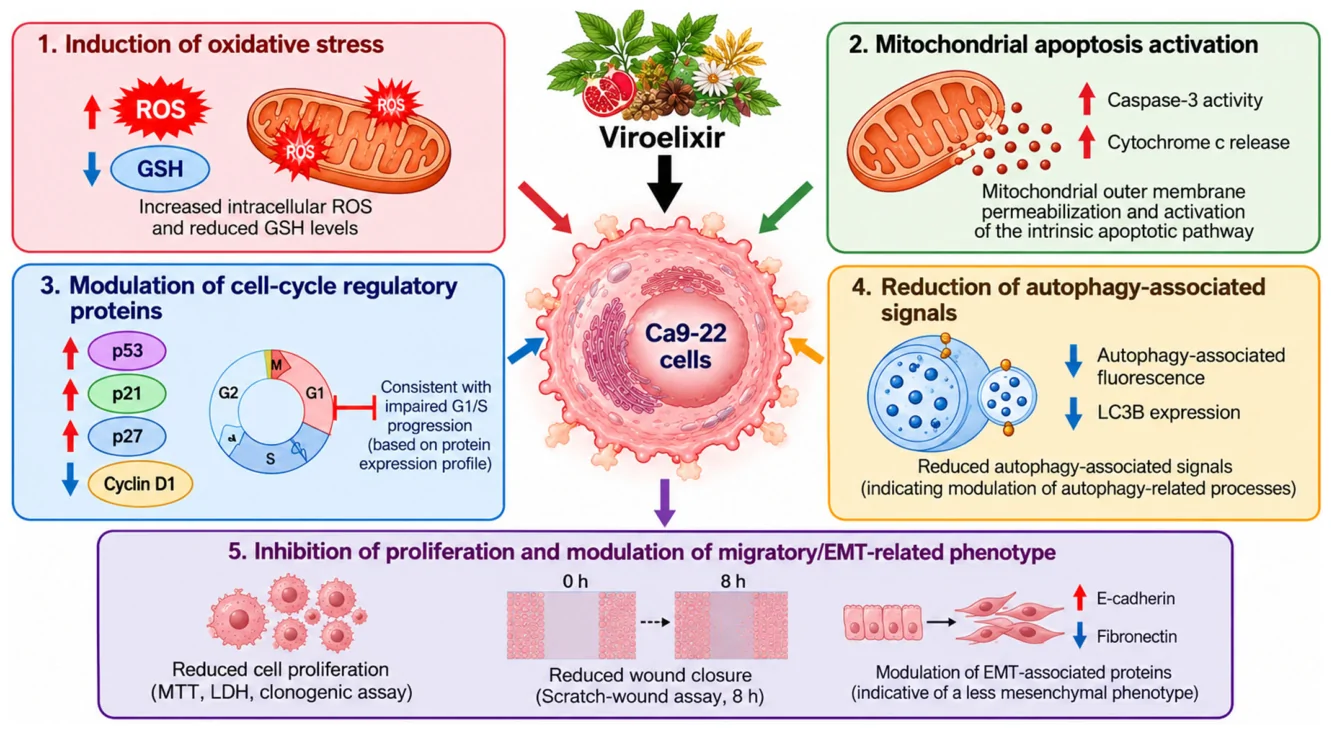
Based on the experimentally observed changes, we propose an integrated model in which the response of Ca9-22 cells to *Viroelixir* is associated with coordinated alterations in cell-cycle regulatory proteins, mitochondrial apoptosis, autophagy-associated signals, redox homeostasis, wound closure/EMT-associated markers, and oncogenic signaling. Red upward arrows indicate an increase, whereas blue downward arrows indicate a decrease in the corresponding markers or cellular responses following *Viroelixir* treatment.

Based on the experimentally observed changes, we propose a working model in which the anticancer activity of *Viroelixir* in Ca9-22 cells is associated with coordinated alterations in cell-cycle regulatory proteins, mitochondrial apoptosis, autophagy-associated signals, redox homeostasis, wound closure/EMT-associated markers, and oncogenic signaling pathways (Figure 9). These findings identify molecular processes potentially involved in the cellular response to *Viroelixir*; however, their individual causal contributions remain to be established through pathway-specific rescue or reactivation experiments. Furthermore, *Viroelixir* enhances the sensitivity of oral cancer cells to conventional chemotherapeutic agents, including cisplatin and 5-fluorouracil.

## 3. Discussion

This study provides in vitro evidence that *Viroelixir*, an aqueous formulation combining green tea- and pomegranate-derived constituents, exerts selective anticancer activity against human gingival squamous cell carcinoma cells through the coordinated modulation of multiple cellular and molecular pathways. *Viroelixir* inhibited Ca9-22 cell proliferation, modulated the expression of key cell-cycle regulatory proteins, induced mitochondrial apoptosis, suppressed autophagy and wound closure, altered oxidative stress, modulated key oncogenic signaling pathways, and enhanced the activity of conventional chemotherapeutic agents. Collectively, these findings support the concept that phytochemical combinations may represent a promising multitargeted therapeutic strategy for the treatment of oral squamous cell carcinoma. One of the most important findings of this study is the selective antiproliferative activity of *Viroelixir* toward oral cancer cells while exerting minimal cytotoxicity against normal oral epithelial cells. Selective cytotoxicity is a highly desirable characteristic for anticancer agents because it may reduce adverse effects commonly associated with conventional chemotherapy. Comparable selective anticancer activities have been reported for several phytochemical-derived compounds including anethole, eugenol, carnosol, and curcumin analogs, which preferentially target malignant cells while sparing normal oral cells [9,10,11,28,29,30,31,32]. The marked reduction in cell viability, increased LDH release, and suppression of clonogenic survival observed in the present study indicate that *Viroelixir* effectively impairs both the short-term viability and the long-term clonogenic potential of Ca9-22 cells.

One of the most notable findings of the present study was the marked difference in *Viroelixir* sensitivity among the oral cell models. Ca9-22 gingival carcinoma cells displayed a pronounced and reproducible response, whereas the two independent tongue carcinoma models, CAL-27 and SCC-9SCC-9, showed limited acute sensitivity. Rather than indicating generalized toxicity toward OSCC cells, these findings identify a cell-line-specific response that may reflect differences in molecular background, cellular origin, redox regulation, or survival signaling. Interestingly, differential treatment responsiveness between these oral carcinoma models has also been observed in our previous studies, in which tongue carcinoma cells showed greater resistance than Ca9-22 cells to several tested compounds [10,11,33]. This supports the biological relevance of the heterogeneous response observed here and suggests that intrinsic cellular characteristics may influence *Viroelixir* sensitivity. Nevertheless, the present findings should not be interpreted as establishing a general difference between gingival and tongue carcinomas, since only one gingival carcinoma cell line was investigated. Comparative studies using larger panels of gingival and tongue OSCC models, together with molecular profiling, will be required to determine whether anatomical origin, molecular background, redox regulation, apoptotic threshold, or survival signaling contributes to this differential sensitivity.

The discrepancy between the relatively preserved short-term MTT activity and the reduced clonogenic capacity of SCC-9SCC-9 cells also suggests that *Viroelixir* may produce cellular effects that extend beyond acute cytotoxicity. Because clonogenic survival reflects the ability of cells to undergo sustained proliferation after treatment, these findings may indicate delayed cytostatic or reproductive effects in SCC-9SCC-9 cells. However, this interpretation requires confirmation using longitudinal viability and cell-cycle analyses.

The antiproliferative effect of *Viroelixir* was associated with significant modulation of key cell-cycle regulators. Notably, *Viroelixir* increased the expression of p53, p21, and p27 while suppressing Cyclin D1 expression. Since p53 is a major upstream regulator of p21 transcription, the concomitant upregulation of both proteins suggests activation of a p53-dependent cell-cycle checkpoint response. This pathway plays a crucial role in preventing uncontrolled cellular proliferation by promoting cell-cycle arrest under conditions of cellular stress. In addition, p27 functions as a cyclin-dependent kinase inhibitor that further reinforces growth arrest. Conversely, Cyclin D1 is a critical regulator of G1/S transition, and its downregulation may limit cyclin D/CDK complex formation, thereby preventing retinoblastoma protein (Rb) phosphorylation and subsequent progression toward DNA synthesis. Collectively, these findings suggest that *Viroelixir* inhibits oral cancer cell proliferation through coordinated regulation of cell-cycle checkpoint proteins, leading to G1-phase cell-cycle arrest. Similar mechanisms have been reported for EGCG, punicalagin, rapamycin, and curcumin-derived compounds in oral cancer and other malignancies [28,29,34,35].

The differential sensitivity observed among oral cancer cell lines may reflect intrinsic differences in genetic background, antioxidant capacity, p53 status, metabolic activity, and activation of survival pathways. Interestingly, Ca9-22 cells appeared markedly more responsive to *Viroelixir* than CAL-27 and SCC-9SCC-9 cells, suggesting that intrinsic molecular characteristics may influence the response to *Viroelixir* treatment.

Another major finding is the ability of *Viroelixir* to induce apoptosis through the intrinsic mitochondrial pathway. Apoptosis induction represents one of the principal mechanisms through which many anticancer agents eliminate malignant cells. In the present study, *Viroelixir* significantly increased apoptotic cell populations while promoting Bax upregulation, Bcl-2 downregulation, cytochrome c release, caspase-9 activation, caspase-3 cleavage, and a reduction in total PARP-1 expression. Together, these molecular changes are consistent with activation of the intrinsic mitochondrial apoptotic pathway through the cytochrome c/caspase cascade. Punicalagin, one of the major pomegranate polyphenols, has been reported to induce Bax upregulation, Bcl-2 downregulation, cytochrome c release and caspase activation through the mitochondrial pathway [27,36]. Likewise, EGCG has been shown to promote apoptosis and inhibit tumor growth through modulation of multiple signaling pathways in several cancer models [23,37,38,39,40]. Therefore, the pro-apoptotic effects observed in this study may result from the complementary actions of bioactive compounds present in both green tea and pomegranate extracts.

*Viroelixir* reduced dye-detected autophagy-associated activity and LC3B expression. These endpoints support an effect on the autophagy pathway, but they do not distinguish reduced autophagosome formation from increased turnover. Confirmation with an autophagic-flux design, such as LC3-II and p62 assessment in the presence and absence of a lysosomal inhibitor, would be required before concluding that *Viroelixir* blocks cytoprotective autophagy. The reduction in autophagy-associated fluorescence together with decreased LC3B expression indicates that *Viroelixir* affects autophagy-related processes in Ca9-22 cells. However, these measurements do not constitute a direct assessment of autophagic flux. In particular, reduced LC3B-associated signals may reflect altered autophagosome formation, turnover, or lysosomal clearance. Future studies examining LC3-I/LC3-II conversion and p62/SQSTM1 in the presence and absence of lysosomal inhibitors will be required to define the precise effect of *Viroelixir* on autophagic flux.

Oxidative stress appears to play a central role in the anticancer activity of *Viroelixir.* The present study demonstrated that *Viroelixir* significantly increased intracellular ROS and mitochondrial superoxide production while simultaneously reducing intracellular glutathione levels. Cancer cells generally maintain elevated antioxidant defenses to counterbalance excessive ROS production and support survival. Therefore, depletion of GSH together with increased ROS accumulation may render oral cancer cells more susceptible to oxidative damage. The marked increase in mitochondrial superoxide production further suggests that mitochondria represent a major target of *Viroelixir*-induced oxidative stress. Excessive mitochondrial ROS generation can promote mitochondrial membrane destabilization, cytochrome c release, caspase activation, and apoptotic cell death. These findings are fully consistent with the observed increase in Bax expression, reduction in Bcl-2 levels, activation of caspase-9 and caspase-3, and induction of apoptosis. Similar ROS-mediated apoptotic mechanisms have been reported for EGCG, punicalagin, and several other polyphenol-rich natural products in oral cancer and other malignancies [29,31,41].

*Viroelixir* reduced wound closure and shifted EMT-associated proteins toward increased E-cadherin and reduced fibronectin. These changes are consistent with diminished motility and a less mesenchymal phenotype. Suppression of ERK1/2, STAT3, AKT1, NF-κB, and p38 phosphorylation may contribute to these effects, but the present data establish associations rather than pathway-specific causality.

The combination experiments provide preliminary evidence that *Viroelixir* can enhance responses to cisplatin and 5-FU in Ca9-22 cells. Estimated CI values supported strong synergy with cisplatin at 1/2000 and 1/1000, whereas 5-FU interactions ranged from variable/additive to moderately synergistic. Because a strict fixed-ratio design was not used, these estimates should be interpreted cautiously. Matrix-based dose–response experiments with formal Chou–Talalay or response-surface analysis, together with toxicity testing in normal oral cells, are needed to establish the magnitude and therapeutic selectivity of these interactions.

In addition to ERK1/2, STAT3, and NF-κB inhibition, *Viroelixir* significantly suppressed AKT1 activation. The PI3K/AKT pathway is one of the most frequently dysregulated signaling cascades in oral cancer and plays a critical role in promoting cell survival, proliferation, migration, EMT, and resistance to chemotherapy [42,43,44]. Accordingly, the observed reduction in AKT1 phosphorylation may be associated with several of the biological responses observed following *Viroelixir* treatment. However, pathway-reactivation or rescue experiments will be required to establish a direct causal contribution.

Combination strategies using natural compounds and conventional chemotherapeutic agents are increasingly being explored to overcome drug resistance and reduce treatment-associated toxicity. Because *Viroelixir* simultaneously modulates cell-cycle regulatory proteins, apoptosis, autophagy, oxidative stress, and multiple survival signaling pathways, it may represent a promising adjuvant candidate capable of improving therapeutic efficacy through complementary mechanisms of action.

An additional emerging direction concerns the potential impact of *Viroelixir* on tumor-derived extracellular vesicles. Because EVs contribute to intercellular communication and can transfer proteins, lipids, and nucleic acids involved in tumor progression and treatment responses, determining whether *Viroelixir* modifies EV release, composition, or biological activity represents an important extension of the present findings. Ongoing work in our laboratory is addressing this question by characterizing the molecular cargo of EVs released from *Viroelixir*-treated oral cancer cells.

## 4. Materials and Methods

### 4.1. Preparation of Viroelixir

*Viroelixir* is a previously described polyphenol-rich formulation derived from green tea and pomegranate peel. Its preparation and phytochemical composition have been reported in our recent studies [26,27]. The formulation contains several classes of bioactive polyphenols, including catechins such as epigallocatechin gallate (EGCG), ellagitannins including punicalagin, ellagic acid, and flavonoids. In the present study, *Viroelixir* was prepared according to the previously described protocol. Briefly, 5 g of green tea and 5 g of pomegranate peel powder were suspended in 100 mL of sterile water. The preparation was centrifuged, and the recovered aqueous phase was passed through a 0.2-micrometer filter and stored at 4 °C until use.

### 4.2. Cell Lines and Culture Conditions

Human gingival squamous cell carcinoma cells (Ca9-22) and tongue squamous cell carcinoma cells (CAL-27 and SCC-9SCC-9) were used as oral cancer models. The non-tumorigenic oral epithelial cell line GMSM-K was used as a normal control. Ca9-22 cells were obtained from the RIKEN BioResource Research Center (Tsukuba, Japan), whereas CAL-27 and SCC-9SCC-9 cells were purchased from ATCC (Manassas, VA, USA). GMSM-K cells were kindly provided by Dr. Daniel Grenier (Université Laval, Québec, QC, Canada). Ca9-22 cells were cultured in RPMI-1640 medium, whereas CAL-27, SCC-9SCC-9 and GMSM-K cells were maintained in DMEM/F12 medium. All media were supplemented with 10% fetal bovine serum (FBS), 0.2% penicillin–streptomycin, and 0.2% Fungizone. Cells were maintained at 37 °C in a humidified atmosphere containing 5% CO_2_.

### 4.3. MTT Cell-Viability Assay

Ca9-22, CAL-27, SCC-9SCC-9, and GMSM-K cells were seeded in 12-well plates and allowed to adhere overnight before exposure to *Viroelixir* (1/10,000 to 1/200) for 24 h. MTT was added to a final concentration of 0.5 mg/mL and incubated for 3 h at 37 °C in the dark. After removal of the medium, formazan crystals were solubilized with 500 μL of 0.4% HCl in isopropanol. Aliquots (100 μL) were transferred in triplicate to a 96-well plate, and absorbance was measured at 550 nm. Viability was calculated as [(OD treated − OD blank)/(OD control − OD blank)] × 100. Untreated cells were defined as 100% viability. Each independent experiment was performed with four technical replicates per experimental condition. The number of independent experiments differed according to the cell model: Ca9-22 cells were analyzed in nine independent experiments (*n* = 9) to confirm the reproducibility of their pronounced response to *Viroelixir*; CAL-27 and SCC-9SCC-9 cells were each analyzed in four independent experiments (n = 4 for each cell line), with SCC-9SCC-9 included as a second tongue squamous cell carcinoma model to assess the consistency of the response observed in CAL-27 cells; and non-tumorigenic GMSM-K cells were analyzed in three independent experiments (n = 3) as a non-malignant oral epithelial comparator.

### 4.4. LDH-Release Assay

Membrane damage was assessed using the Cytotoxicity Detection Kit (LDH; Roche Diagnostics, Mannheim, Germany Cat. No. 11644793001) according to the manufacturer’s instructions and as previously described [10,11,29]. After 24 h of *Viroelixir* exposure, 50 µL in triplicate for each supernatant was placed in a 96-well plate, and 50 µL of solution mix consisting of 1.25 µL of catalysis solution and 56.25 µL of dye solution was added to the supernatant in the dark until color development; then the plate was read at 490 nm. The LDH activity was measured using the following formula: % of LDH activity = [treated cells (absorbance) − negative control (absorbance)]/[positive control (absorbance) − negative control (absorbance)] × 100. The data were represented by percentage. Absorbance at 490 nm for cells treated with 1/100 of Triton corresponds to 100% cytotoxicity. The experiment was repeated more than 6 times.

### 4.5. Morphological Assessment

Cell morphology was examined by phase-contrast light microscopy after 24 h of *Viroelixir* exposure, and representative images were acquired with a Nikon COOLPIX 995 digital camera (Nikon Corporation, Tokyo, Japan). Three independent experiments were performed.

### 4.6. Clonogenic Assay

As described by Semlali et al. [29,31], cells (2 × 10^3^ per well) were exposed to the indicated *Viroelixir* dilutions and maintained for 14 days, with medium replacement every 2 days. Colonies were washed with PBS, fixed with cold methanol for 10 min, stained with 1% crystal violet for 10 min, washed, and imaged. Four independent experiments were performed.

### 4.7. Apoptosis Assay

Apoptosis was evaluated 24 h after *Viroelixir* treatment using the Annexin V/Propidium Iodide Apoptosis Detection Kit (BioLegend, San Diego, CA, USA #640932), as previously described [28,31,33,36]. Briefly, all cells were plated in 6-well plates at a density of 4 × 10^4^ cells/well and allowed to adhere overnight. The following day, cells were treated with different *Viroelixir* dilutions for 24 h, then trypsinized and collected by centrifugation. The cells were subsequently stained with Annexin V-FITC/Propidium Iodide according to the manufacturer’s protocol. All analyses were performed by the BD Accuri™ C6 Plus Flow Cytometer (BD Biosciences, San Jose, CA, USA). The total events were classified as viable (AnxV−/PI−), early apoptotic (AnxV+/PI−), late apoptotic (AnxV+/PI+), and necrotic cells (AnxV−/PI+). The experiment was repeated five times.

### 4.8. Autophagy Assay

Autophagic activity was assessed using a fluorescent autophagy detection kit followed by flow-cytometric analysis. Briefly, Ca9-22 and CAL-27 cells were seeded into 6-well plates at a density of 4 × 10^4^ cells per well and allowed to adhere overnight. Cells were then treated with various dilutions of *Viroelixir* for 24 h. Following treatment, cells were resuspended in 500 μL of culture medium containing 1/5 Red staining reagent and incubated for 60 min at 37 °C in the dark. Cells were subsequently collected by low-speed centrifugation and washed three times with 1× assay buffer. Flow-cytometric analysis was performed using a BD Accuri™ C6 Plus Flow Cytometer [10,28]. The experiment was repeated four times.

### 4.9. Oxidative Stress Assays

Intracellular reactive oxygen species (ROS) levels were measured using the Intracellular Total ROS Activity Assay Kit (ImmunoChemistry Technologies, Davis, CA, USA) according to the manufacturer’s recommendations. Briefly, cells were seeded in 6-well plates at a density of 4 × 10^4^ cells per well and allowed to adhere overnight. Following treatment with *Viroelixir* for 24 h, cells were incubated with the Total ROS Green reagent for 1 h at 37 °C in the dark, washed, and analyzed using a BD Accuri™ C6 Plus Flow Cytometer [9,11,36]. Fluorescence intensity was measured on the FL1 channel.

### 4.10. Mitochondrial Superoxide

Production was evaluated using MitoSOX™ Red Mitochondrial Superoxide Indicator (Invitrogen, Thermo Fisher Scientific Waltham, MA, USA) according to the manufacturer’s recommendations [33]. After treatment with *Viroelixir* for 24 h, cells were incubated with MitoSOX™ Red reagent for 10 min at 37 °C in the dark, washed with PBS, and immediately analyzed by flow cytometry. Fluorescence was detected on the FL2 channel.

### 4.11. Intracellular Glutathione (GSH)

GSH levels were quantified using the Intracellular GSH Assay Kit (ImmunoChemistry Technologies, USA) according to the manufacturer’s protocol. Following treatment, cells were incubated with the GSH detection reagent, washed with assay buffer, and analyzed by flow cytometry [10,11,29,33,34]. Fluorescence intensity was measured using the FL1 channel. All experiments were performed using a BD Accuri™ C6 Plus Flow Cytometer and repeated at least four times.

### 4.12. Intracellular Protein Expression Analysis

The expression of proteins involved in apoptosis, cell-cycle regulation, and intracellular signaling pathways, including Bax, Bcl-2, p21, p27, p53, Cyclin D1, phosphor-NF-κB p65 (Ser529), and phosphor-AKT1 (Ser473), were analyzed by flow cytometry. Cells were seeded at a density of 1 × 10^6^ cells per condition and allowed to adhere for 24 h before treatment [11,33,36]. Following 24 h of *Viroelixir* exposure, cells were washed with PBS, detached using trypsin, collected into flow-cytometry tubes, and centrifuged at 500× *g* for 5 min. Cells were washed once with PBS and fixed with 4% paraformaldehyde for 20 min at room temperature. After fixation, cells were washed and permeabilized for 30 min using PBS containing 2% fetal bovine serum (FBS) and 0.2% Triton X-100. Cells were then incubated for 30 min at room temperature in blocking buffer consisting of PBS supplemented with 5% FBS. For Bax, Bcl-2, p21, p27, p53, and Cyclin D1 detection, primary antibodies (Table 1) were diluted 1:200 in PBS containing 2% FBS and 0.05% Triton X-100 and incubated overnight at 4 °C. Cells were subsequently washed twice and incubated for 1 h at room temperature in the dark with FITC-conjugated anti-mouse or anti-rabbit secondary antibodies (Table 2) diluted 1:100.

For phospho-NF-κB p65 (PE-conjugated) and phospho-AKT1 (APC-conjugated), cells were incubated directly with the antibodies for 1 h at room temperature in the dark without secondary antibody staining. Following staining, cells were washed twice, resuspended in 200 μL PBS containing 2% FBS, and analyzed using a BD Accuri™ C6 Plus Flow Cytometer. Results were expressed as mean fluorescence intensity (MFI) or percentage of positive cells.

For each experimental condition, unstained controls consisting of cells processed identically but without the specific antibody were included to determine baseline autofluorescence and background fluorescence. Background fluorescence was taken into account during flow-cytometric analysis to minimize the contribution of baseline autofluorescence and non-antibody-associated background signal to the phospho-specific fluorescence measurements.

For phosphoprotein analyses, each independent experiment was normalized to its corresponding untreated control, which was defined as 100%. *Viroelixir*-treated values were expressed relative to the corresponding untreated control within the same experiment. Consequently, following normalization, all untreated control values were equal to 100%, resulting in a normalized control mean of 100% with an SD of 0.

### 4.13. Wound-Healing Assay

As described by Semlali et al. [29,34], the wound-closing capacity of Ca9-22 cells in the presence of different *Viroelixir* dilutions was assessed using a scratch-wound assay. Images were acquired immediately after scratching (0 h) and after 8 h of treatment. Wound dimensions were quantified using ImageJ software (version 1.47v), and wound closure at 8 h was calculated relative to the initial wound measurement at 0 h. The 8 h time point was selected to evaluate early changes in wound closure while limiting the contribution of prolonged treatment-associated changes in cell number. Representative images and quantitative data are presented in Figure 6.

### 4.14. Statistical Analysis

Data are presented as mean +/− SD from independent experiments; sample sizes are specified in the corresponding figures or legends. Analyses were performed using GraphPad Prism 9.0. Depending on the experimental design, two-group comparisons used an unpaired two-tailed Student’s *T*-test, whereas experiments with multiple groups used one-way or two-way ANOVA followed by an appropriate multiplicity-adjusted post hoc test. *p* < 0.05 was considered statistically significant.

### 4.15. Exploratory Drug-Interaction Analysis

Combination-index (CI) values were calculated as exploratory estimates of drug interaction. CI values < 1, =1, and >1 conventionally indicate synergistic, additive, and antagonistic interactions, respectively. However, because the present experiments were not designed using a strict fixed-ratio or comprehensive dose-matrix approach, the calculated CI values were not considered definitive pharmacological evidence of synergy, additivity, or antagonism. The primary analysis therefore focused on whether *Viroelixir* enhanced the antiproliferative effects of cisplatin and 5-FU.

## 5. Conclusions

*Viroelixir* exhibited a pronounced and reproducible cell-line-specific anticancer response in vitro, with Ca9-22 gingival carcinoma cells displaying substantially greater sensitivity than the other oral cell models tested. In Ca9-22 cells, treatment was associated with changes in cell-cycle regulatory proteins, mitochondrial apoptosis, autophagy-associated readouts, redox homeostasis, wound closure, EMT-associated proteins, and the phosphorylation status of multiple oncogenic signaling proteins. *Viroelixir* also enhanced the antiproliferative effects of cisplatin and 5-fluorouracil, although definitive pharmacological synergy requires validation using an appropriate fixed-ratio or dose-matrix design. Collectively, these findings support further investigation of *Viroelixir* and of the molecular determinants underlying the pronounced sensitivity of Ca9-22 gingival carcinoma cells.

## Figures and Tables

**Table 1:** The following primary antibodies were used for flow cytometry.

Target Protein	Clone	Host	Supplier	Catalog Number	Dilution
Bax	B-9	Mouse	Santa Cruz, Biotechnology Dallas, TX, USA	sc-7480	1:200
Bcl-2	100	Mouse	Santa Cruz Biotechnology, Dallas, TX, USA	sc-509	1:200
PARP-1	F-2	Mouse	Santa Cruz Biotechnology, Dallas, TX, USA	sc-8007	1:200
Caspase-3	3CSP03	Mouse	Santa Cruz Biotechnology, Dallas, TX, USA	sc-56046	1:200
Caspase-9	F-7	Mouse	Santa Cruz Biotechnology, Dallas, TX, USA	sc-17784	1:200
p53	Bp53-12	Mouse	Santa Cruz Biotechnology, Dallas, TX, USA	sc-263	1:200
p21	F-5	Mouse	Santa Cruz Biotechnology, Dallas, TX, USA	sc-6246	1:200
p27	O.N.54	Mouse	Santa Cruz Biotechnology, Dallas, TX, USA	sc-71813	1:200
Cyclin D1	A-12	Mouse	Santa Cruz Biotechnology, Dallas, TX, USA	sc-8396	1:200
LC3B	Rabbit mAb	Rabbit	Cell Signaling Technology, Danvers, MA, USA	#2775S	1:200
Phospho-STAT3 (Tyr705)	XP Rabbit mAb	Rabbit	Cell Signaling Technology, Danvers, MA, USA	#9145S	1:200
Phospho-ERK1/2 (Thr202/Tyr204)	Rabbit mAb	Rabbit	Cell Signaling Technology, Danvers, MA, USA	#4377S	1:200
Phospho-p38 MAPK (Thr180/Tyr182)	Rabbit mAb	Rabbit	Cell Signaling Technology, Danvers, MA, USA	#9211S	1:200
Cleaved Caspase-3 (Asp175)	Rabbit mAb	Rabbit	Cell Signaling Technology, Danvers, MA, USA	#9661S	1:200
Cleaved Caspase-9 (Asp315)	Rabbit mAb	Rabbit	Cell Signaling Technology, Danvers, MA, USA	#9505S	1:200
Phospho-NF-κB p65 (Ser529)	B33B4WP	Mouse	Invitrogen/eBioscience, San Diego, CA, USA	12-9863-42	5:100
Phospho-AKT1 (Ser473)	SDRNR	Mouse	Invitrogen/eBioscience, San Diego, CA, USA	17-9715-42	5:100
E-cadherin	clone G-10	Mouse	Santa Cruz, Biotechnology, Dallas, TX, USA	sc-8426	1:200
Fibronectin	Mouse monoclonal	Mouse	Santa Cruz, Biotechnology, Dallas, TX, USA	sc-18827	1:200

**Table 2:** The following secondary antibodies were used for flow cytometry.

Secondary Antibody	Host	Fluorochrome	Supplier	Catalog Number	Dilution
Goat Anti-Rabbit IgG (H+L)	Rabbit	Alexa Fluor™ 488	Invitrogen	R37116	1:100
Goat Anti-Mouse IgG (H+L) F(ab’)_2_ Fragment	Mouse	FITC	Merck Millipore, Burlington, MA, USA	AQ303F	1:100

## Data Availability

All data generated or analyzed during this study are included in this article. Raw data can be made available by the corresponding author upon reasonable request.

## Abbreviations

5-FU	5-Fluorouracil
AKT	Protein kinase B
ANOVA	Analysis of Variance
CI	Combination Index
DMEM	Dulbecco’s Modified Eagle Medium
DMSO	Dimethyl Sulfoxide
ECL	Enhanced Chemiluminescence
EGCG	Epigallocatechin-3-gallate
EMT	Epithelial–Mesenchymal Transition
ERK	Extracellular Signal-Regulated Kinase
FBS	Fetal Bovine Serum
FITC	Fluorescein Isothiocyanate
GSH	Glutathione
LDH	Lactate Dehydrogenase
NF-κB,	Nuclear Factor Kappa B
OSCC	Oral Squamous Cell Carcinoma
PBS	Phosphate-Buffered Saline
PI	Propidium Iodide
pP38	Phosphorylated p38 mitogen-activated protein kinase
ROS	Reactive Oxygen Species
SDS	Sodium Dodecyl Sulfate
SEM	Standard Error of the Mean
STAT3	Signal Transducer and Activator of Transcription 3
